# Disseminated Cutaneous Mycobacterium chelonae Infection in an Immunocompetent Patient

**DOI:** 10.7759/cureus.43170

**Published:** 2023-08-08

**Authors:** Jesus Ivan Martinez-Ortega, Felipe de Jesus Perez-Hernandez, Marco Antonio Rodriguez-Castellanos, Elvis Martinez-Jaramillo, Jorge Carlos Guillermo-Herrera

**Affiliations:** 1 Department of Dermatology, Dermatological Institute of Jalisco, "Dr. Jose Barba Rubio", Zapopan, MEX; 2 Department of Internal Medicine, High Specialty Regional Hospital of the Yucatan Peninsula, Merida, MEX; 3 Department of Pathology, Faculty of Medicine and Health Science, McGill University, Montreal, CAN; 4 Department of Research, High Specialty Regional Hospital of the Yucatan Peninsula, Merida, MEX

**Keywords:** atypical mycobacteria, infection, immunocompetent, disseminated cutaneous, mycobacterium chelonae

## Abstract

We present a case report on disseminated cutaneous *Mycobacterium chelonae* infection with a sporotrichoid pattern in an immunocompetent patient. The aim of this report is to contribute to the existing knowledge on the clinical presentation and management of this uncommon presentation.

## Introduction

*Mycobacterium chelonae* is a rapidly growing form of nontuberculous mycobacteria and is typically an uncommon cause of cutaneous infections [1]. The sporotrichoid pattern associated with mycobacterial infections is not commonly observed in immunocompetent hosts, nor is the disseminated form. However, in immunosuppressed patients, the infection may have a progressive course with an increasing number of lesions and an extended time until clearance [2,3]. We present a case of cutaneous disseminated *M. chelonae* infection in an immunocompetent patient that persisted for a full decade.

## Case presentation

**Figure 1:**
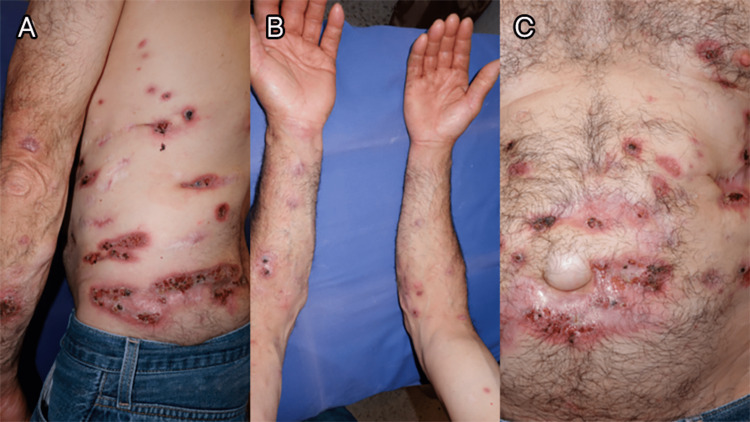
Clinical images Clinical lesions on the skin scattered on the trunk (A), arms (B), and abdomen (C)

A 50-year-old male with no significant past medical history presented with a 10-year history of asymptomatic skin lesions. He was a farmer from a rural zone of Jalisco, Mexico. Any other recreational water exposures were denied. Physical examination revealed violaceous, friable, ulcerated nodules ranging in size from 1×1 cm to 7×22 cm, scattered bilaterally on the upper extremities, trunk, and abdomen (Figure 1).

**Figure 2:**
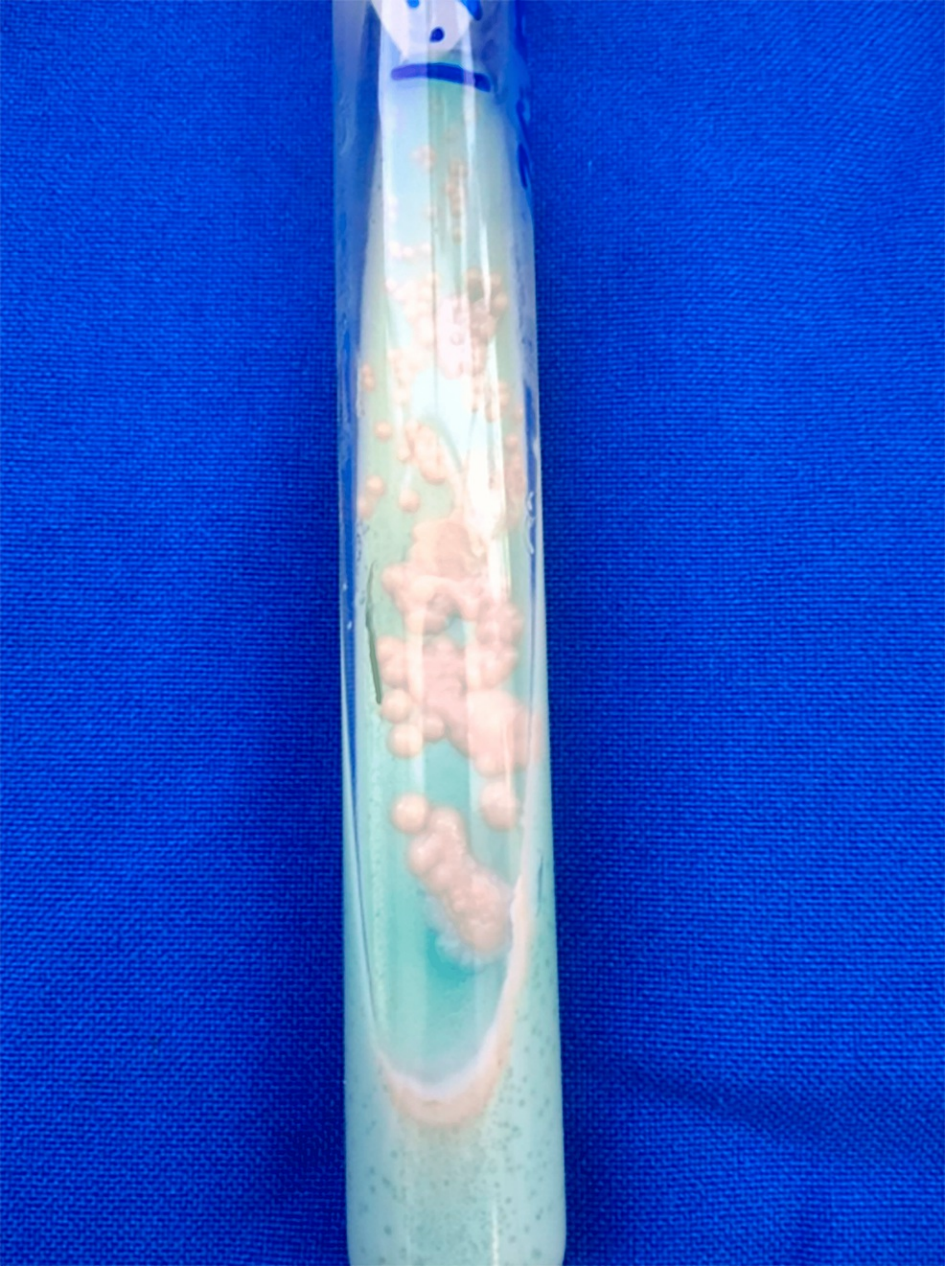
Culture The culture shows the presence of rapidly growing mycobacteria colonies

Punch skin biopsies showed nonspecific granulomatous inflammation in the dermis. Stains yielded negative results, but the Löwenstein-Jensen culture demonstrated the presence of rapidly growing mycobacteria (Figure 2).

Fungal and bacterial culture tests were negative. HIV and diabetes mellitus screening tests were negative, and other laboratory tests did not reveal any significant alterations. Polymerase chain reaction (PCR) analysis confirmed the causative agent as *Mycobacterium chelonae*. Based on the clinical presentation and confirmed diagnosis, the patient received a four-month course of clarithromycin, doxycycline, and ciprofloxacin with improvement of skin lesions.

## Discussion

*Mycobacterium chelonae* is typically an uncommon cause of cutaneous infections. The sporotrichoid pattern and disseminated form observed in our patient are unusual manifestations [2,3]. Moreover, it has been found that most of these extended lesions are developed in immunocompromised patients [4]. Wallace et al. found 100 specimens over 10 years of *Mycobacterium chelonae* infections, 72 cases were found with immunosuppression, and 62 were linked to corticosteroid use. Additionally, common risk factors for the infection included trauma and medical procedures. Although our patient did not recall any trauma, the considerable time frame of 10 years and the inherent occupational risk of being a farmer suggest that the development of these extended lesions might be attributed to one or multiple traumas over the course of days or even months with some source of infection leading to this particular clinical presentation [5]. Interestingly, there have been reports of apparently spontaneous and disseminated cutaneous infections of *Mycobacterium chelonae* in seemingly immunocompetent patients. However, it is important to note that in this reported case, the patient was an 86-year-old female, and immunosenescence may explain the dissemination of the infection [6,7]. Additionally, encountering such long-lasting evolution of lesions is highly infrequent. One case with eight years of lasting lesions surrounded by puckered scars (like the present case) indicated an abnormal injury-healing process that led to fibrosis and tissue retraction. Remarkably, this case involved a widespread cutaneous infection in a 40-year-old healthy female without any preexisting immunosuppression [8].

The sporotrichoid-like spread of cutaneous *Mycobacterium chelonae* has also been reported previously, and as the disseminated cutaneous lesions, it appears to be more frequent in immunocompromised patients [9]. Recently, Uslu et al. reported five cases of cutaneous infection by *Mycobacterium chelonae*, and although three of the five patients were apparently immunocompetent, they were all over 85 years old [10]; as mentioned earlier, we believed that immunosenescence should be considered as part of the immunocompromised concept [7].

It is worth noting that single-gene inborn errors of immunity may cause Mendelian susceptibility to mycobacterial disease, leading to the development of mycobacterial infections in otherwise healthy individuals. Thirteen genes have been reported so far, and their products are involved in interferon (IFN)-γ-dependent antimycobacterial immunity [11]. In light of this, we speculate that some apparent cases reported in immunocompetent patients may present immunosenescence, while others, such as our case, could potentially be underdiagnosed cases of Mendelian susceptibility to mycobacterial diseases. Further research and investigation are needed to fully understand the underlying mechanisms and immunological factors contributing to these infections in both immunocompetent and immunocompromised individuals.

This highlights the uniqueness of the clinical presentation in our case. The prolonged persistence of the infection for a decade in an immunocompetent patient further underscores the atypical nature of the condition. Our findings contribute to the current understanding of varied presentations of *Mycobacterium chelonae* infections on the skin, emphasizing the need for vigilance and deepening the understanding of the immune response to *Mycobacterium chelonae*.

## Conclusions

We report a rare case of disseminated cutaneous *Mycobacterium chelonae* infection with a sporotrichoid pattern in an immunocompetent patient. The clinical presentation observed in this case is distinct from the typical manifestations and highlights the importance of considering atypical presentations in the diagnosis and management of cutaneous mycobacterial infections. Our findings provide valuable clinical insights and contribute to the existing literature on this uncommon presentation.
